# Comparison of three rebound tonometers in normal and glaucomatous dogs

**DOI:** 10.1111/vop.13043

**Published:** 2022-11-28

**Authors:** Kimberly J. Hodgson, Christine D. Harman, Shayla Bajric, Ava Cabble, Amanda L. Anderson, Hemalatha Palanivel, David A. Taylor, András M. Komáromy

**Affiliations:** ^1^ Department of Small Animal Clinical Sciences, College of Veterinary Medicine Michigan State University East Lansing USA; ^2^ Reichert Technologies New York USA

**Keywords:** *ADAMTS10*, canine, central corneal thickness (CCT), glaucoma, intraocular pressure (IOP), rebound tonometry

## Abstract

**Objective:**

The objectives of the study were to compare intraocular pressure (IOP) readings across a wide range and obtained via three rebound tonometers in *ADAMTS10*‐mutant Beagle‐derived dogs with different stages of open‐angle glaucoma (OAG) and normal control dogs and to investigate the effect of central corneal thickness (CCT).

**Animals Studied:**

Measurements were performed on 99 eyes from 50 Beagle‐derived dogs with variable genetics—16 non‐glaucomatous and 34 with *ADAMTS10*‐OAG. Seventeen OAG eyes were measured twice—with and without the use of IOP‐lowering medications.

**Procedures:**

IOP was measured in each eye using three tonometers with their “dog” setting—ICare® Tonovet (TV), ICare® Tonovet Plus® (TVP), and the novel Reichert® Tono‐Vera® Vet (TVA)—in randomized order. CCT was measured with the Accutome® PachPen. Statistical analyses included one‐way ANOVA, Tukey pairwise comparisons, and regression analyses of tonometer readings and pairwise IOP‐CCT Pearson correlations (MiniTab®).

**Results:**

A total of 116 IOP measurements were taken with each of the three tonometers. When comparing readings over a range of ~7–77 mmHg, mean IOPs from the TV were significantly lower compared with TVP (−4.6 mmHg, *p* < .001) and TVA (−3.7 mmHg, *p* = .001). We found no significant differences between TVA and TVP measurements (*p* = .695). There was a moderate positive correlation between CCT and IOP for TVA (*r* = 0.53, *p* < .001), TVP (*r* = 0.48, *p* < .001), and TV (*r* = 0.47, *p* < .001).

**Conclusions:**

Our data demonstrate strong agreement between TVP and TVA, suggesting that the TVA may similarly reflect true IOP values in canines. CCT influenced IOP measurements of all three tonometers.

## 1 INTRODUCTION

Accurate and reliable methods to obtain intraocular pressure (IOP) are critical to diagnosing and monitoring glaucoma and other ocular conditions.^1^ Glaucoma is a leading cause of irreversible blindness in humans and many veterinary species, including dogs.^1^, ^2^, ^3^, ^4^, ^5^, ^6^, ^7^, ^8^ Canine glaucoma, a disorder that involves the irreversible death of retinal ganglion cells and progressive vision loss, is associated with elevated IOP.^1^, ^3^ Indirect tonometry is used routinely to measure IOP noninvasively in clinical settings, with applanation and rebound tonometers being applied most commonly in veterinary ophthalmology.^9^

**FIGURE 1:**
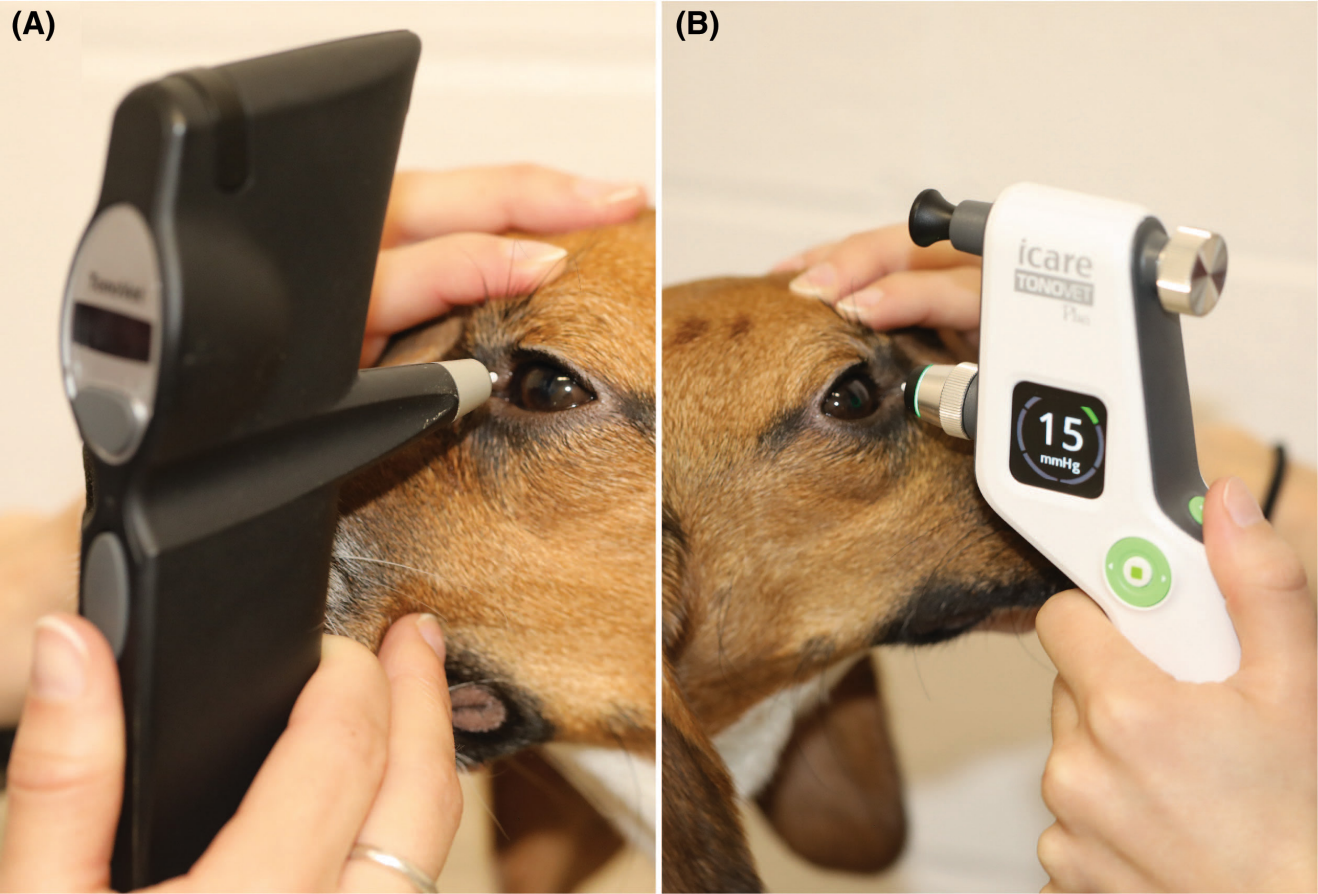
Use of the iCare rebound tonometers TonoVet® (A) and TonoVet Plus® (B) in dogs

Rebound tonometers propel an electromagnetic probe against the surface of the cornea and use the velocity of the probe as it impacts the cornea to determine IOP.^9^, ^10^ This technique can be performed without the use of ocular surface anesthesia. TonoVet® (TV; Icare Finland Oy) and TonoVet Plus® (TVP; Icare Finland Oy) are two popular rebound tonometers currently on the market for use in animals (Figure 1). Their accuracy was studied extensively in dogs and other species and compared to manometry and applanation tonometers in vivo and ex vivo.^8^, ^9^, ^11^, ^12^, ^13^, ^14^, ^15^, ^16^, ^17^, ^18^, ^19^, ^20^, ^21^, ^22^, ^23^ Numerous variables were shown to affect the IOP measurements, including the probe's angle, cornea‐to‐probe distance, and central corneal thickness (CCT).^17^, ^22^, ^23^, ^24^, ^25^, ^26^

**FIGURE 2:**
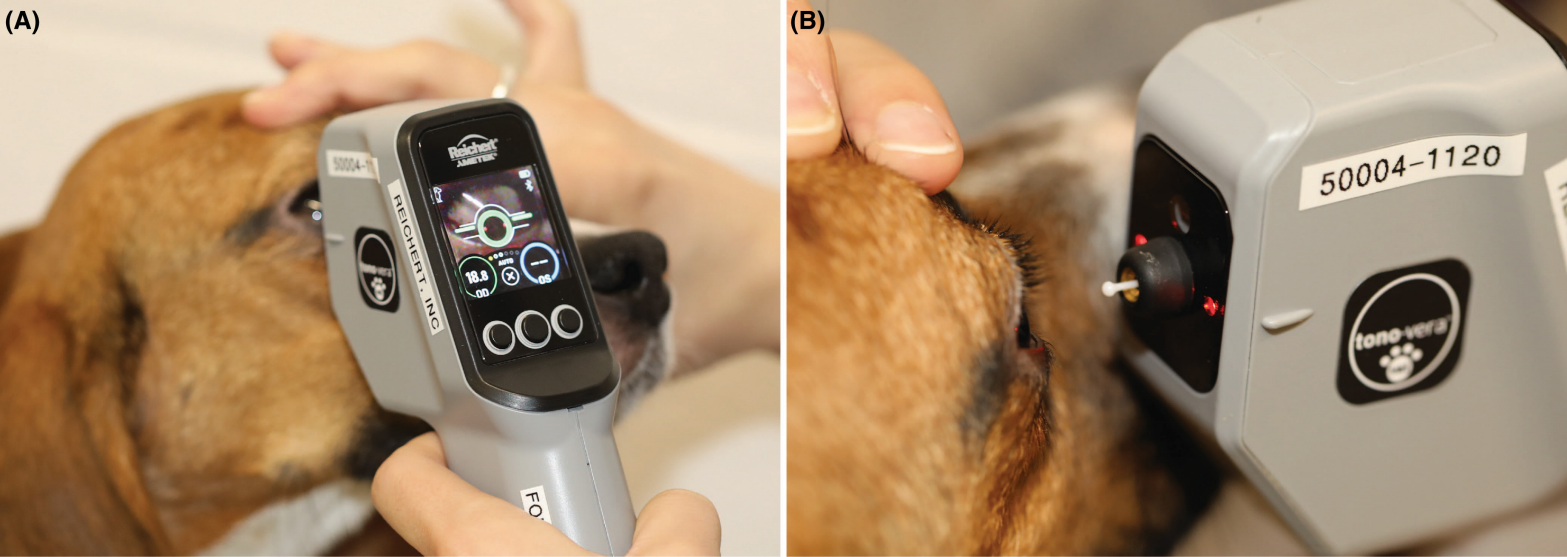
Use of the Reichert rebound tonometer Tono‐Vera® in dogs. (A) A ring on the display changes from red to green when the instrument is correctly aligned with the apex of the cornea. (B) Two red LED lights, one on each side of the electromagnetic probe, measure the alignment with the corneal surface

Recently, a third rebound tonometer, the Tono‐Vera® Vet (TVA; Reichert Technologies), was introduced for use in animals (Figure 2). While there are numerous published studies on the use of TV and TVP in animals,^8^, ^9^, ^11^, ^12^, ^13^, ^14^, ^15^, ^16^, ^17^, ^18^, ^19^, ^20^, ^21^, ^22^, ^23^, ^24^, ^25^, ^26^ to the best of our knowledge, no such studies exist for the TVA. This study aimed to compare TV, TVP, and TVA rebound tonometers across a range of IOPs in purpose‐bred normal Beagle‐derived dogs and dogs with *ADAMTS10*‐open‐angle glaucoma (*ADAMTS10*‐OAG). In addition, we studied the effect of CCT on IOP readings across the three rebound tonometers.

## 2 METHODS

### 2.1 Animals

Ninety‐nine eyes from 50 purpose‐bred, Beagle‐derived dogs were used in this study: 34 dogs were homozygous for the G661R missense mutation in the *ADAMTS10* gene and at different stages of *ADAMST10*‐OAG.^27^ The remaining 16 dogs were non‐glaucomatous with two unaffected carriers of the *ADAMTS10* mutation, one achromatopsia‐affected and homozygous for a *CNGB3* genomic deletion mutation (*CNGB3* ^*−/−*^),^28^ and 13 *wild types* regarding the *ADAMTS10* and *CNGB3* mutations. Genotypes were confirmed based on *ADAMTS10* and *CNGB3* gene sequences.^27^, ^28^ The median age of the dogs was 2.5 years, with a range of 0.3–11.7 years. The sex distribution was 30 females and 20 males. The dogs were group‐housed in the same environment at Michigan State University College of Veterinary Medicine with a 12‐hour light and 12‐hour dark cycle and fed the same diet. The studies complied with the Association for Research in Vision and Ophthalmology (ARVO) Statement for the Use of Animals in Ophthalmic and Vision Research and were approved by the Michigan State University Institutional Animal Care and Use Committee (IACUC).

### 2.2 Tonometry

Intraocular pressures were measured in all eyes with the three rebound tonometers TV, TVP, and TVA. All the data was collected over the course of 2 weeks and between 7 Am and 11 Am. Since diurnal rebound tonometry by use of TV is routinely performed as part of dog colony management and specific IOP studies,^29^, ^30^ all animals were well adjusted to the experimental setup. The nine *ADAMTS10*‐OAG‐affected dogs (17 eyes) receiving IOP‐lowering glaucoma medications had their IOPs taken twice—once on normal dosing and once with a skipped dose—resulting in 116 measurements from 99 eyes. The second readings were done to obtain naturally occurring high IOPs. Potential discomfort from elevated IOP was preemptively treated with gabapentin (10–20 mg/kg PO q8 hr; Ascend Laboratories, LLC). The potential IOP‐lowering effect of gabapentin did not interfere with the study design,^31^ since measurements with the three tonometers occurred at the same time under the same medical treatment conditions.

All measurements were taken by a single investigator (KJH). The three tonometers were calibrated by the manufacturers less than 1 year before the study and were all used with the “dog” setting. Computer‐generated randomization was used to select the order of the three tonometers for each eye and the order of the eyes (right vs. left eye) for each dog as follows: The tonometers and eyes were numbered and their order assigned by use of the web‐based Google random number generator (Google, LLC). One IOP reading was obtained with each tonometer according to the manufacturers' manuals.^32^, ^33^, ^34^ The number of measurements for a single IOP reading differed between instruments: six readings were taken by TV and TVP, with the displayed result being the average of four—the highest and lowest readings were discarded. The TVA has two modes, either 1 measurement or 3+ measurements. We used the 3+ measurements mode: A minimum of three measurements were taken with additional measurements being added (up to six total) if any of them were more than 10% different from the median of the measurements taken. The final IOP reading was based on the three measurements that were within the 10% tolerance. If six measurements were made and this criterion was not satisfied according to the built‐in on‐screen indicator, the measurements were repeated.

All three tonometers have feedback mechanisms to ensure proper measurement distance and centration. With the TV, this is achieved by the “quality” of the beep upon each measurement. If there is an erroneous measurement, the TV beeps twice, instead of one short beep, and shows an error message on the display. The display on the side of the TVP, an upgraded version of the TV, indicates if the probe was too far or too close to the cornea upon each measurement, and a red/green LED indicator light at the probe base indicates if the instrument is within the required tilt angle for measurement (Figure 1B). TVP measurements can only be taken if the device is within the necessary tilt‐angle range (approximately +5 to −10 degrees). The preferred distance to the cornea is 4–8 mm for both TV and TVP. The ActiView™ positioning system of the TVA uses a complementary metal oxide semiconductor (CMOS) camera and two LED lights to actively guide the operator to the correct alignment (Figure 2): The display on the back of the instrument indicates the proper distance (6 mm ± 1.5 mm) and centration over the apex of the cornea. In addition, displayed probe angle indicators help the operator to keep the probe angle within the required +/− 15 degrees of horizontal. By default, the TVA automatically takes and averages three readings upon proper XYZ alignment of the instrument without the need to push any buttons. If the device detects a high standard deviation upon the first three measurements, it will continue to take readings up to a maximum of six.

### 2.3 Pachymetry

The Accutome® PachPen was used for CCT measurement in each eye following administration of ocular surface anesthesia (proparacaine HCl 0.5% ophthalmic solution; Akorn, Inc.) and lubrication (OptixCare® Plus Eye Lube; CLCMEDICA). The PachPen® takes nine readings and displays the average measurement. All measurements were taken by a single investigator (CDH) following tonometry.

### 2.4 Statistics

Statistical analyses were performed with Minitab® (Minitab® Inc). A *p*‐Value of <.05 was considered significant. The IOP measurements obtained with the three tonometers were compared with one‐way ANOVA, Tukey pairwise comparisons, Bland–Altman plots, and regression analyses. The association between IOP and CCT was determined separately for each of the three tonometers with regression analyses.

## 3 RESULTS

### 3.1 Comparison of IOP measurements

**FIGURE 3:**
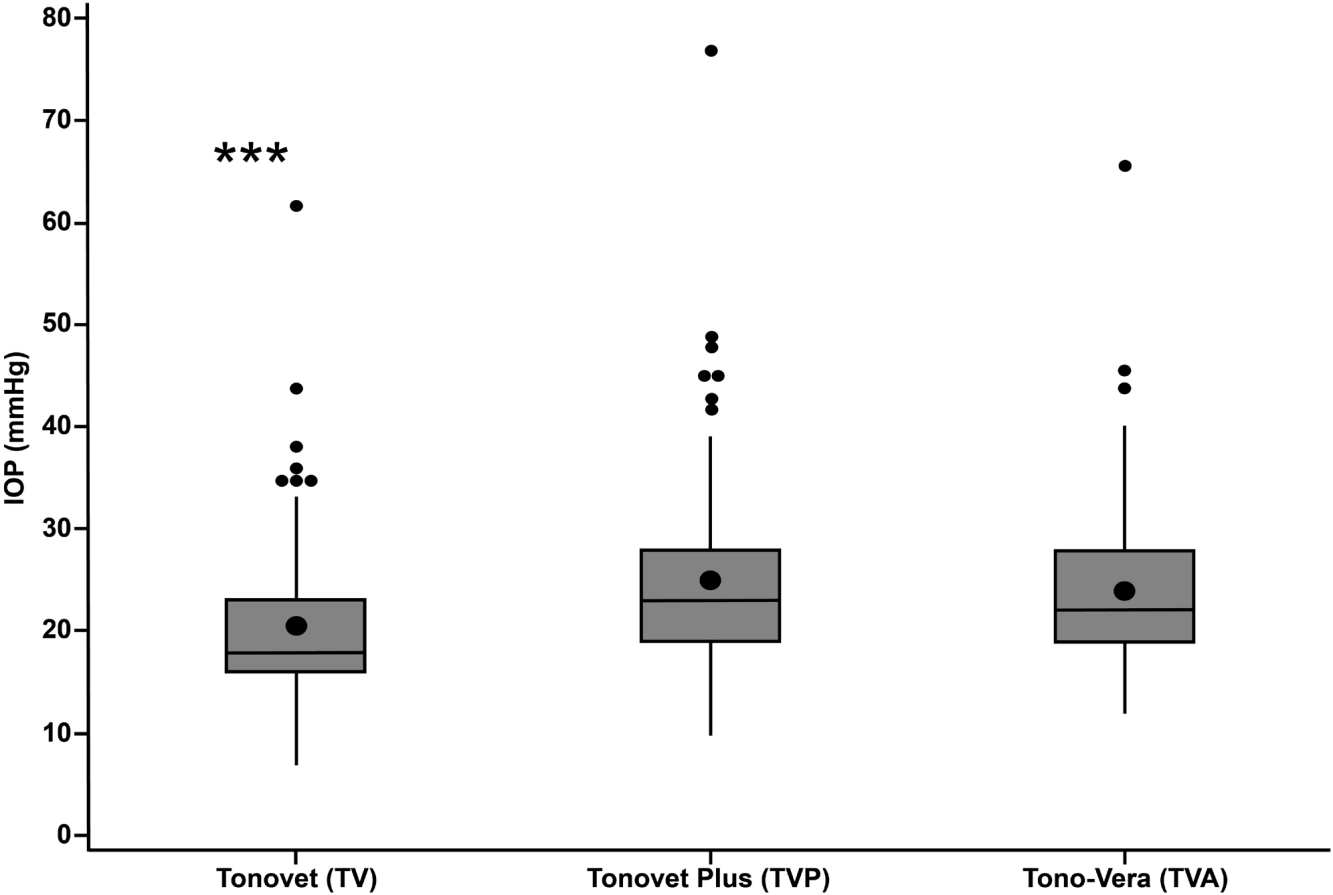
Boxplots summarizing IOPs measured with three rebound tonometers. Overall means (+/− SD) were 20.4 ± 7.5 mmHg (TV), 24.9 ± 8.9 mmHg (TVP), and 24.1 ± 7.7 mmHg (TVA). There was a significant difference between mean IOPs, with TV values being lowest (***, *p* < .001); there was no significant difference between TVP and TVA measurements. The boxes represent the middle 50% of data, with the centerline being the median. The bottom and top whiskers show the first and third quartile, respectively. Outliers are shown as small dots. The larger black dot inside the box represents the mean.

**FIGURE 4:**
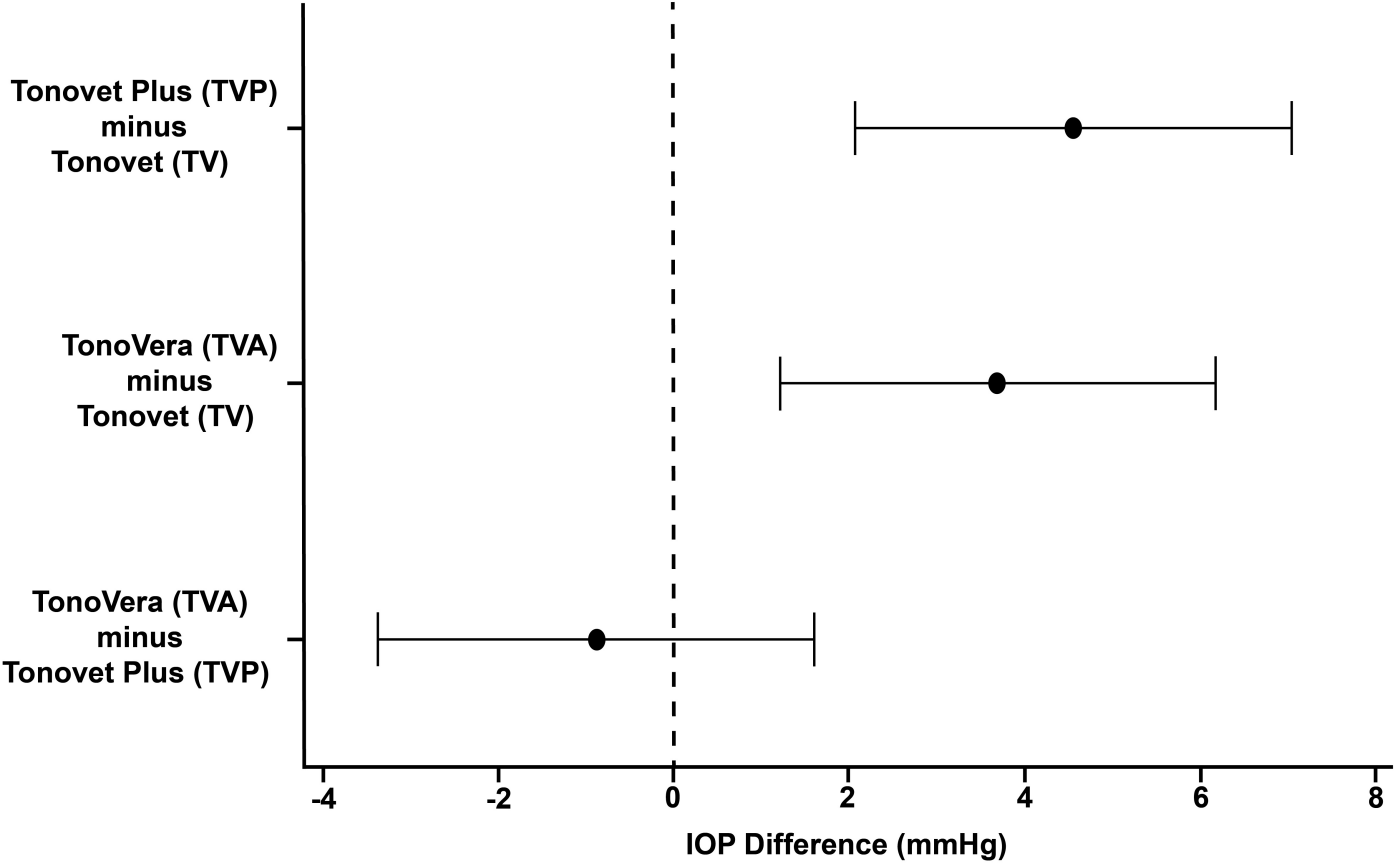
Tukey simultaneous tests for differences of IOP means. The black dots represent mean differences between rebound tonometers. Because the 95% confidence intervals (CI; horizontal lines) do not contain zero (vertical dashed line), the corresponding means are significantly different for TVP‐TV (4.56 ± 1.06 mmHg; CI: 2.08, 7.04) and TVA‐TV (3.7 ± 1.06 mmHg; CI: 1.22, 6.18), respectively (*p* ≤ .001). There was no significant difference between TVA and TVP means (−0.86 ± 1.06 mmHg; CI: −3.34, 1.62; *p* = .695).

**FIGURE 5:**
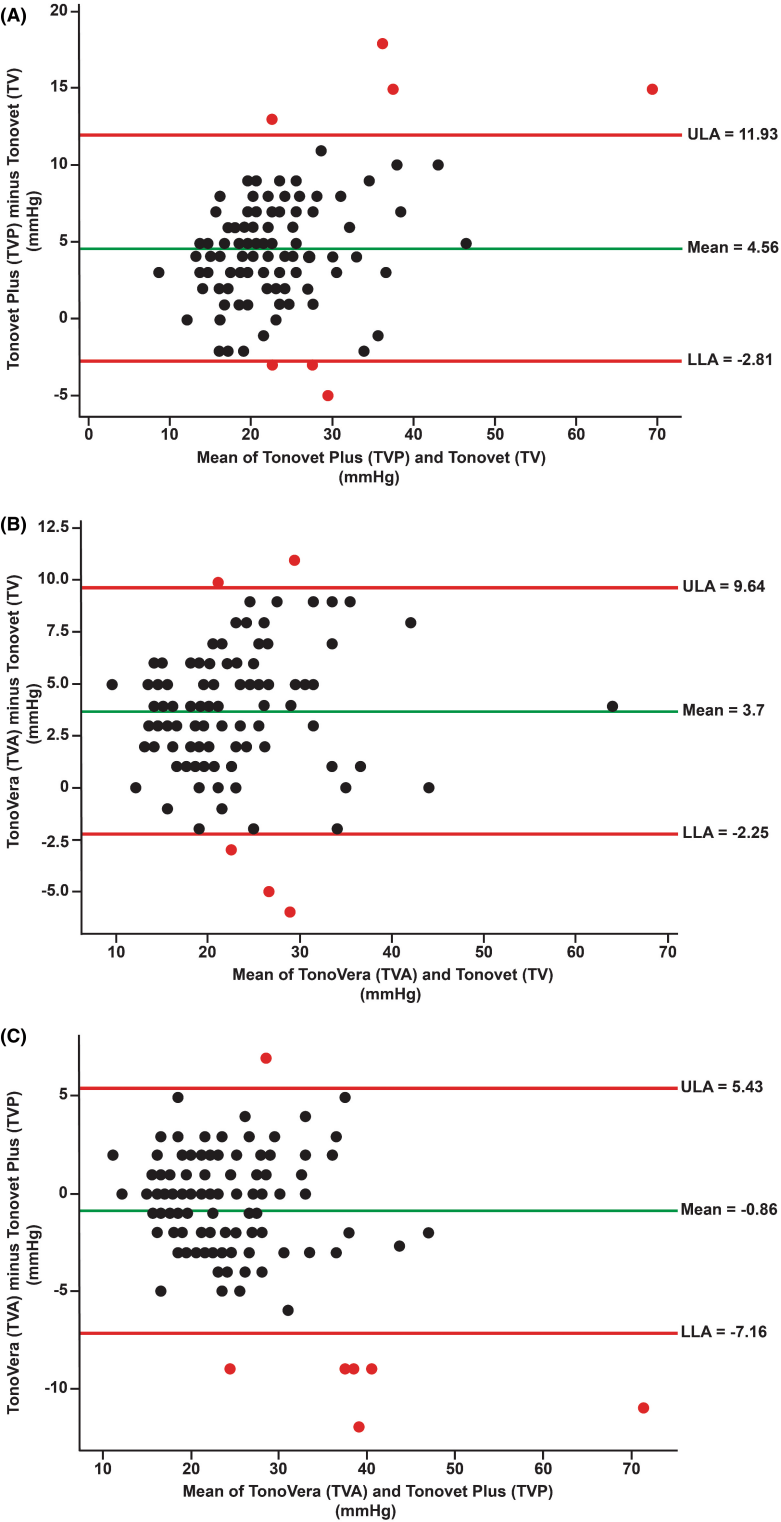
Bland–Altman plots comparing three rebound tonometers. The estimated means of the differences between two tonometers are shown by the green horizontal lines. The 95% CI is marked by the red lines representing the upper (ULA) and lower (LLA) limits of agreement (mean ± 1.96 SD). Overall, the IOP measurements did not follow any trends and were mainly located within the 95% CI. The estimated means of the differences (±SD) were 4.56 ± 3.76 mmHg for TVP‐TV (A), 3.7 ± 3.03 mmHg for TVA‐TV (B), and − 0.86 ± 3.21 mmHg for TVA‐TVP (C).

**FIGURE 6:**
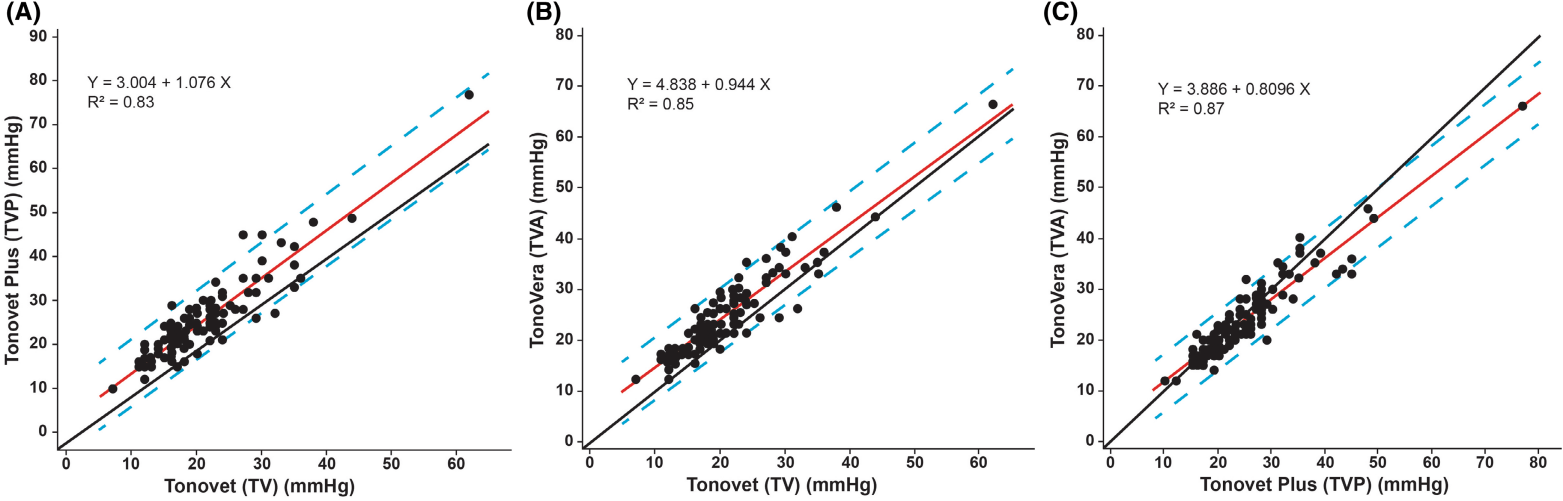
IOP regression analyses between tonometers with linear equations and R^2^ values. The red lines show strong linear correlation between TV and TVP (A), TV and TVA (B), and TVP and TVA (C) with the blue dashed lines marking the 95% prediction intervals. Compared to the black y = x line, TVP and TVA showed the best match (C); in contrast, TV underestimated most IOPs when compared to TVP (A) and TVA (B), respectively, with data points mostly located to the left of the y = x line.

The IOP data from the right (ranges: TV, 11–62 mmHg; TVP, 12–77 mmHg; TVA, 12–66 mmHg) and left eyes (ranges: TV, 7–44 mmHg; TVP, 10–49 mmHg; TVA, 12–46 mmHg) showed no statistical differences between them for all three tonometers (*p* > .05). Mean IOP values ± SD measured by TV (20.4 ± 7.5 mmHg) were significantly lower (*p* < .001) than those measured with TVP (24.9 ± 8.9 mmHg) and TVA (24.1 ± 7.7 mmHg), respectively (Figures 3, 4). We found no significant differences between mean IOPs measured with TVP and TVA (*p* = .695). Bland–Altman analyses showed close agreements between the three tonometers without trends (Figure 5). Linear regression analyses showed good agreement between the three tonometers with strong R^2^ values between 0.82 and 0.87 (Figure 6). We observed the most comparable agreement with the highest R^2^ value of 0.87 between TVA and TVP (Figure 6C). The graphs show that TV underestimated most TVA and TVP readings (Figures 6A,B).

### 3.2 Effects of CCT on IOP measurements

**FIGURE 7:**
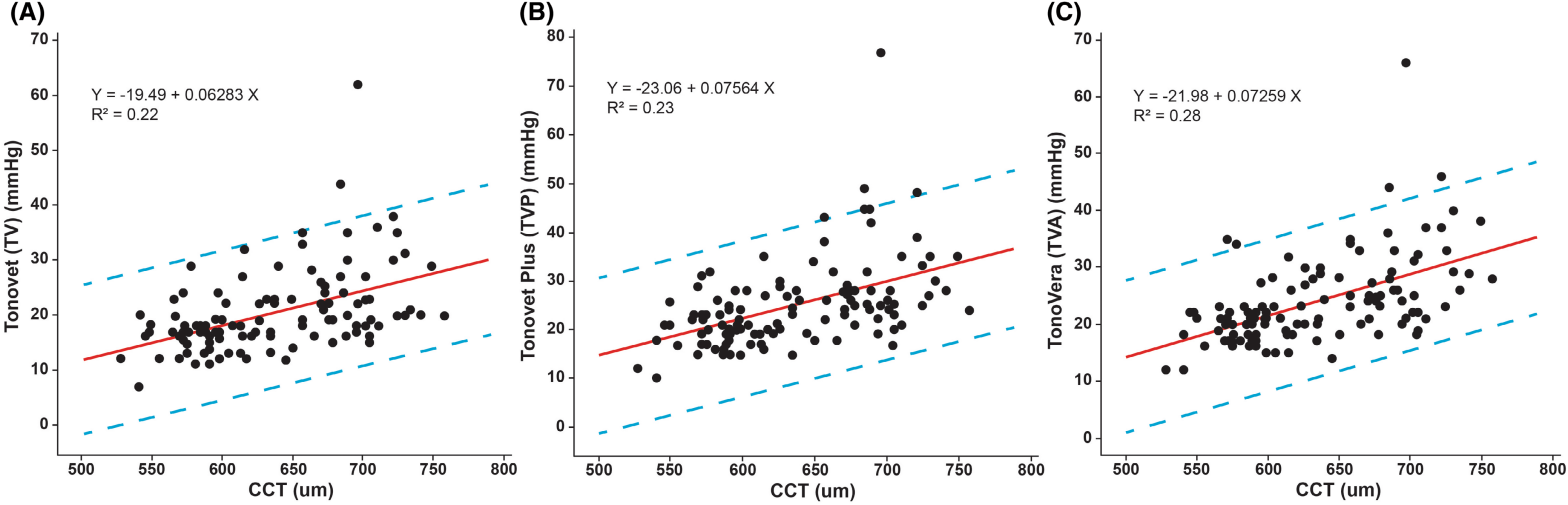
Regression analyses between CCT and IOP with linear equations and R^2^ values for TV (A), TVP (B), and TVA (C). The blue dashed lines mark the 95% prediction intervals. Even though the R^2^ values were low, the positive linear relationships between CCT and IOP were significant for all three rebound tonometers (*p* < .005).

Mean CCT ± SD was 634 ± 57 μm (range: 527–758 μm). Linear regression analyses showed that IOP measurements taken with all three rebound tonometers were significantly and positively correlated with CCT (Figure 7): TVA (*r* = 0.53, *p* < .001), TVP (*r* = 0.48, *p* < .001), and TV (*r* = 0.47, *p* < .001). High IOP spikes, shown as outliers on the graphs, were not associated with corneal thickening.

## 4 DISCUSSION

We showed good agreement between IOP readings taken with the three rebound tonometers TV, TVP, and TVA. There were no significant differences in IOP means between TVP and TVA. The IOP means were 3.7–4.6 mmHg lower when comparing TV to TVA and TVP, respectively. While comparisons of TV and TVP readings to each other and true manometric IOPs were published before for dogs and other species and showed strong linear correlation,^11^, ^13^, ^16^, ^19^, ^20^, ^21^, ^22^ to the best of our knowledge, this is the first study evaluating the TVA in dogs. Previously, both TV and TVP rebound tonometers were shown to underestimate true IOP in canine eyes, but they were more accurate than applanation tonometers.^11^, ^13^, ^16^, ^20^, ^21^ The clinical importance of IOP underestimation, especially with the TV at higher IOPs, has been previously emphasized by others.^16^, ^20^, ^21^ A recent study in canine eyes showed that the TVP underestimated IOPs much less than other tonometers, including the TV.^20^ Even though we did not include manometric IOPs in our study, the close agreement of TVA with the previously studied TVP suggests that TVA readings closely represent true canine IOPs with some degree of underestimation, especially with higher IOPs ≥50 mmHg.^20^

The more sophisticated alignment mechanisms included in TVP and TVA provide more accurate rebound tonometry readings since IOP measurements are affected by both probe angulation, corneal region (central vs. peripheral), and probe‐cornea distance.^17^, ^24^ While these alignment systems did not inhibit the process of data collection in our well‐trained dogs, in our clinical experience, they can complicate and delay data gathering in less cooperative canine patients since it takes more time to align the tonometers. The less sophisticated alignment system allows more efficient IOP measurements in such animals with the TV. Still, it may lead to inaccurate or variable results due to less precise positioning upon measurement. In contrast to the TVP, the alignment system can be turned off on the TVA, allowing more efficient but potentially less accurate IOP measurements without variability assessment.

In addition to the more sophisticated alignment systems, TVP and TVA are calibrated for four animal species: dog, cat, rabbit, and horse.^33^, ^34^ In contrast, the TV has only two defined settings: dog/cat (do) and horse (ho); the third setting “P” is undefined.^31^

Consistent with previous rebound and applanation tonometry studies in dogs,^25^, ^26^ we showed a moderate positive correlation between CCT and IOP readings for all three tonometers tested. Thus, CCT should be considered when taking IOP readings, especially when the corneal thickness is affected by disease. Similar relationships between CCT and IOP have been described in humans,^35^, ^36^, ^37^, ^38^ but they stand in contrast to a few canine data published by others who did not find significant correlations.^23^, ^39^

Limitations of this study include a small sample size (50 dogs) with fewer high‐pressure readings (9) and the omission of manometric IOP. Larger sample sizes, including different canine breeds, could be considered to compare the new TVA with other tonometers more extensively. In addition, a direct comparison of TVA readings with manometric IOPs will be needed. Based on previous manometry‐TVP comparisons and the close TVP‐TVA agreement, we anticipate that the TVA will compare similarly to manometric IOP as the TVP.^20^ Comparisons of the TVA with other methods in additional animal species are also needed.

In conclusion, this study found that IOP measurements taken with TVP and TVA were similar to each other but different from TV IOPs. Thus, the same tonometer should be used for all IOP readings when monitoring individual dogs over time. Nevertheless, the differences between rebound tonometers do not appear to be clinically relevant. We also found that CCT had a significant effect on IOP readings taken by all three rebound tonometers.

## CONFLICT OF INTEREST

DA Taylor and H Palanivel are employees of Reichert® Technologies, and AM Komáromy is a consultant. The company provided the ICare® Tonovet Plus® and Reichert® Tono‐Vera™ to AM Komáromy for the study. AM Komáromy received research funding from PolyActiva Pty. Ltd. and CRISPR Therapeutics while the presented work was conducted. While AM Komáromy also serves as Editor‐in‐Chief of Veterinary Ophthalmology, he was not involved in the review of this manuscript. All other authors declare no conflict of interest.
